# Electrochemical, surface analysis, computational and anticorrosive studies of novel di-imine Schiff base on X65 steel surface

**DOI:** 10.1038/s41598-023-37321-8

**Published:** 2023-06-28

**Authors:** Ahmed. Nasser, M. A. Migahed, N. M. EL Basiony, H. M. Abd-El-Bary, Tarek A. Mohamed

**Affiliations:** 1 The Higher Institute of Engineering, New Elmarg, El-Qalyubia Egypt; 2 Egyptian Petroleum Research Institute, Nasr City, 11727 Cairo Egypt; 3 School of Chemical Engineering, Sungkyunkwan University, Suwon, 16419 Republic of Korea; 4 Department of Chemistry, Faculty of Science (Men’s Campus), Al-Azhar University, Nasr City, 11884 Cairo Egypt

**Keywords:** Chemistry, Electrochemistry

## Abstract

The inhibitory effect of di-imine-SB namely ((*N*^*1*^*Z, N*^*4*^*E)-N*^*1*^*, N*^*4*^-bis (4 (dimethylamino) benzylidene) butane 1,4-diamine) on X65-steel in 1 M HCl has been investigated experimentally and theoretically. The electrochemical impedance spectroscopy (EIS), potentiodynamic polarization (PDP), and weight loss outcomes display the anticorrosion properties of “di-imine- SB”. The inhibitory efficiency exceeds 90% at the optimal concentration of 1 × 10^–3^ M “di-imine- SB”. The metal surface was examined further using scanning electron microscope (SEM) and energy dispersive X-ray (EDX). The effectiveness of the di-imine-SB is returned into its adsorption on X65-steel surface and found in agreement with Langmuir adsorption isotherm. According to the standard Gibbs free energy of adsorption $({\Delta G}_{ads}^{^\circ })$, di-imine-SB adsorption tends to be chemical rather than physical, it increases the activation energy (${\mathrm{E}}_{a}$) of metal dissolution reaction and makes it hard to occur. The PDP data suggested anodic and cathodic type of the di-imine-SB inhibitor. Meanwhile, increasing the resistance of X65-steel to 301 Ω cm^2^ after adding 1 mM of di-imine-SB confirms its protective effect. Whereas, the positive value of the fraction of electron transference (ΔN, 0.746), confirms the affinity of di-imine-SB to share electrons to the partially filed 3d-orbital of Fe forming strong protective film over X65-steel surface. Aided by Monte Carlo (MC) simulation, the calculated adsorption energy (E_ads_) suggests excessive adsorption affinity of di-imine-SB on metal surface over the corrosive chlorides and hydronium ions. A good correlation between the theoretical hypothesis and the experimental inhibition efficiency has been achieved. The comparative study showed the superior of the di-imine-SB as potential corrosion inhibitor compared with those reported before. Finally, global reactivity descriptors; electron affinity (*A*), ionization potential (*I*), electronegativity (*χ*), dipole moment (*µ*), global hardness ($\eta$), electrophilicity index and, Fukui indices were also calculated and found well correlated to the reactivity of di-imine-SB.

## Introduction

Carbon steel is thought to be vulnerable to corrosion in aggressive acidic media, like chloride and sulphate ions^1,2^ which makes it favorable economically and industrially. Corrosion inhibitors, the corresponding safeguards, are therefore deemed essential to either regulate/control or minimize/reduce corrosion rate, particularly in HCl media^3–6^. Organic Schiff bases are considered effective “corrosion inhibitors” of metals/alloys, they have hetero atoms (such as nitrogen, oxygen, sulphur, and/or phosphorous) and π bond(s)^7–10^. Nevertheless, Schiff bases are typically made from inexpensive precursors via the condensation–NH_2_ moiety with either ketone or an aldehyde^11–13^, thus became popular. The imine (–C=N–) functional group(s) and the available electron densities (ED) are essential for Schiff bases to cling/adhere to both metal and/or alloy surfaces. Such adsorption declare the formation of a fluffy/persistent film on steel surfaces, which causes a sever retardation of anodic and/or cathodic reaction(s), that slows down the corrosion rate consequently^14,15^. The innovation of imine metal chelates including benzylidene derivatives have demonstrated excellent efficacy in hindering the corrosion of copper- mild-, carbon- and stainless-steel in HCl, HNO_3_ and H_2_SO_4_ media^16–21^. To effectively apply for X65-steel corrosion inhibitor, a Schiff base with two benzylidene ring(s) and four nitrogen hetero atom(s) has been synthesized in the current study. Employing literature review, the di-imine-SB named ((*N*^*1*^*Z, N*^*4*^*E)-N*^*1*^*, N*^*4*^-bis (4-(dimethyl amino) benzylidene) butane-1,4-diamine), was neither theoretically nor electrochemically considered as a corrosion inhibitor for carbon steel. As a result, it was synthesized and subsequently examined herein as a potential inhibitor of the corrosion of X65-steel in 1 M HCl at various concentrations and temperatures. We have used weight loss, PDP, and EIS approaches to achieve high inhibition efficiency (IE%) on the X65-steel surface. The surface morphology of X65-steel before and after being immersed in 1 M HCl was examined using SEM and EDX methods. Additionally, we have used Materials Studio 6.0 software with the methods of Density Function Theory (DFT) and Monte Carlo (MC) adsorption locator modules to simulate the adsorption of di-imine-SB/X65-steel. The molecular and electronic structures were refined/optimized, and the calculation of E_HOMO_, E_LUMO_, and E_gap,_ allows estimating the inhibition probability of di-imine-SB. It is worth mentioning that, “di-imine-SB” surfactants were found effective anticorrosion in our recent studies^22^ and its chelate efficiency with various metals could be one of our future objectives.

## Experimental

### Chemicals and Schiff base

Chemicals of high purity grade were used as supplied from Alpha Chemical Company including, 37% HCl, Ethanol (≥ 95%). The candidate inhibitor "di-imine-SB" (*N*^*1*^*Z, N*^*4*^*E)-N*^*1*^*,N*^*4*^*-bis* (4(dimethyl-amino)benzylidene)butane1,4-diamine), Supplementary Fig. S1, has been already prepared and fruitfully characterized using elemental analysis, infrared-, Raman- and NMR- spectroscopic measurements by our group^23^.

### Medium test and Steel specimen

The following aqueous solutions were prepared using bi-distilled water at 298 ± 1 K. Then a stock solution of 1 × 10^–2^ M of di-imine-SB was prepared HCl (1 M), followed by additional “di-imine-SB” concentrations (1 × 10^–5^–1 × 10^–3^ M) in HCl (1 M). The elemental analysis of X65-steel in weight percentages (wt.%) are, “98.569 (Fe), 0.210 (Si), 0.103 (C), 0.024 (P), 0.012 (Ni), 1.05 (Mn) and 0.032 (Co)”.

### Weight loss measurements

The weight loss (WL) technique is commonly used for assessing the inhibitor efficiency (IE%)^24^. Several concentrations of di-imine-SB (1 × 10^–5^–1 × 10^–3^ M) in HCl (1 M) were prepared to account for its inhibition efficiency towards X65-steel surface. The WL was carried out in a water bath equipped with a thermostat from 298 to 328 K. The X65-steel coupons were cut into the appropriate dimensions of (4.7 × 1.8 × 0.14) cm^3^ for length, width, and thickness, respectively. After that, it was polished with emery paper (grades 600–2500), rinsed with bi-distilled water, dipped in acetone to check for the complete removal of any oxide coatings, and then dried. The weight of these coupons is weighted up to four decimal places before being immersed in closed glass vessels filled by 100 mL of 1 M HCl (blank solution), and the above-mentioned sequential concentrations of di-imine-SB for 6 h at 98 ± 1 K. To eliminate the corrosion product(s), steel coupons were cleaned, rinsed with bi-distilled water, dried and finally weighed. Each testing was triplicated to measure the average weight loss.

### Electrochemical measurements (ECM)

A 100 mL jacketed glass cell with a platinum sheet “an auxiliary electrode”, Ag/AgCl (3 M KCl) “a reference electrode”, and X65 steel “a working electrode”, all coupled to an electrochemical system that is injected into a capillary tube. A computerised Auto-lab (PGSTAT-128N) potentiostat/galvanostat was used to perform the ECM at first. The working electrode was initially cut into a cylindrical shape, insulated by epoxy resin, and left with a surface area of 1 cm^2^ in contact with solution. Like weight loss coupons, the electrode surface is abraded firmly up to mirror image, rinsed with bi-distilled water and dried. To account for the potential inhibitory behaviour of “di-imine-SB”, the ECM were initially performed on an X65-steel electrode bathed in HCl (1 M) in absence and presence of different concentration of di-imine-SB for 30 min, this period is found enough to reach a steady state of the E_ocp_ (open circuit potential)^25^, see Supplementary Fig. S2. The EIS and PDP measurements were carried out, Consequently, the EIS was performed using frequency ranged from (100–0.0001) kHz with peak-to-peak amplitude of 0.01 V. We have assembled the anodic/cathodic polarization curves within a potential range of ± 400 mV around the E_ocp_ employing 0.001 V/s (scan rate) using NOVA 2.1.4 software and the PDP data outcomes. The weight loss and electrochemical tests were repeated three times, the mean values and standard deviations were given in Supplementary Table S1.

### Surface characterization

QUANTA FEG-250, Field Emission Gun Scanning Electron Microscope (FE-SEM) attached with Energy Dispersive X-ray (EDX) unit has been used for surface characterizations of X65-steel specified slides (1 × 1 × 0.1) cm^3^ in absence (blank) and presence of di-imine-SB at optimum concentration (C_inh, opt_: 1 × 10^–3^ M) after being immersed for 6 h (WL conditions).

### Computational procedure

As the corrosion process investigated herein in acidic media, the examined di-imine-SB molecules in the gaseous- (isolated molecule in vacuum), solvated-, (aqueous, dielectric constant of water is 78.56) and protonated- (HCl) states were optimized and their quantum chemical indices were estimated using DFT utilizing BIOVIA Materials Studio 6.0 (17.1.0.48) software^26^, https://www.3ds.com/products-services/biovia/products/molecular-modeling-simulation/biovia-materials-studio. The DMol3 module has been created using the Generalized Gradient Approximation (GGA) and group functional basis designed to take into consideration its chemical reactivity using medium quality tolerance parameters, a Becke One Parameter (BOP) and Double Numerical plus Polarization (DNP-3.5). The energies (*E*_HOMO_ and *E*_LOMO_) of the Highest Occupied Molecular Orbital (HOMO) and that of the Lowest Unoccupied Molecular Orbital (LUMO), energy gap in eV (ΔE = E_LUMO_ − E_HOMO_), ionization potential (*I*), dipole moment (*µ*), electron affinity (*A*), electronegativity (*χ*) and fraction of the electron transferred (*∆N*) were computed. Aided by MCs computations using the adsorption locator module in vacuum/solvent, simulating the adsorption and interaction between di-imine-SB and X65-steel “Fe (1 1 0)” surface with excellent surface stability^27^. Using the same surface cleavage, the adsorption energy (*E*_ads_) is simulated in a box with dimensions (49.6 × 49.6 × 24.05) Å^3^ with periodic boundary conditions extending the cleavage plane to a (15 × 15) super cell. After that, 15 Å vacuum slab built over Fe (1 1 0) plane to eliminate the periodic boundary effect. The MCs annealing was processed for di-imine-SB over Fe (1 1 0) in gas in which (200H_2_O + 5H_3_O^+^ + 5Cl^−^) acidic solution was simulated through 10 cycles (15,000 steps) for each run, and the *E*_tot_ of the system has been estimated at equilibrium. The force field was set to COMPASS (Condensed-phase Optimized Molecular Potentials for Atomistic Simulation Studies) with fine quality of the energy calculation^28^.

## Results and discussion

### Weight loss measurements, effect of concentration and temperature

**Figure 1:**
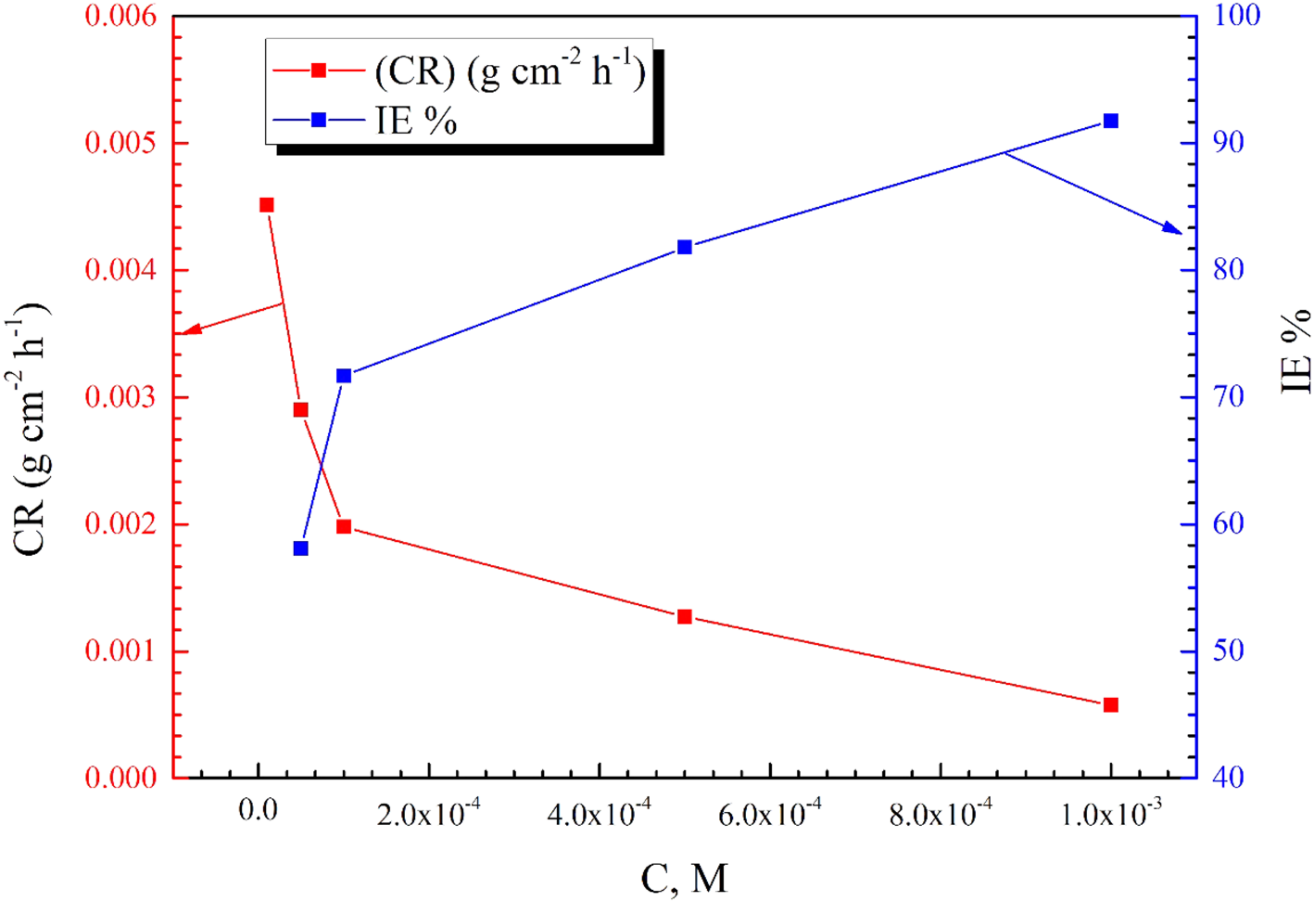
Influence of the inhibitor dose on the rate of corrosion (CR) and the inhibition performance (*IE%*) of the prepared di-imine-SB inhibitor at 298 ± 1 K.

The weight loss (WL) output parameters of X65-steel in the absence/presence of di-imine-SB/HCl (1 M) at 298 ± 1 K are displayed in Fig. 1 and Table 1 implementing different concentrations of an inhibitor. The corrosion rate (*CR*) in g cm^−2^ h^−1^ were calculated by implementing the formula: $\mathrm{CR }=\frac{\mathrm{\Delta W}}{\mathrm{S d t}}$ , where (S) is the surface area of the steel coupons (cm^2^), (d) is the density of iron (7.85 g cm^−3^) and (t) is the immersion time (6 h). Whereas, the degree of surface coverage $\left(\theta \right)$ and inhibition efficiency ($\mathrm{IE }\%$) values can be calculated from: ${\mathrm{IE }\%=\theta \times100=\left(\frac{\Delta \mathrm{W}}{{\mathrm{W}}_{0}}\right)\times 100}$, where, ΔW is average weight loss (${\mathrm{W}}_{0}-\mathrm{W}$), W_0_ and W are the weight loss of X65-steel coupons in the absence and presence of di-imine-SB inhibitor, respectively. Due to the adsorption of inhibitor molecules on coupons surface^29^, IE% rises and the corrosion rate (CR) falls upon increasing C_inh_ (Fig. 1 and Table 1). Maximum IE of 92% is achieved with 1 × 10^–3^ M Schiff base at 298 ± 1 K owing to the molecular interactions beside the inductive effect (+ I) of −(Me)_2_ groups which enhances the ED at nitrogen’s leading to effective adsorption^30^.

**Table 1:** Corrosion parameters obtained from weight loss (WL) measurements for X65-steel coupons after 6 h immersions in 1 M HCl adding different concentrations of di-imine-SB and without adding it at different temperatures.

Temperature ± 1 K	C (M)	1 M HCl Medium				
		ΔW (g)	CR		Ɵ	IE (%)
			(g cm^−2^ h^−1^)	(g m^−2^ d^−1^)		
298	Blank	0.7873	0.0069	1656	–	–
	1 × 10^–5^	0.4286	0.0038	912	0.4556	45.56
	5 × 10^–5^	0.3300	0.0029	696	0.5808	58.08
	1 × 10^–4^	0.2229	0.0019	456	0.7168	71.68
	5 × 10^–4^	0.1435	0.0012	288	0.8177	81.77
	1 × 10^–3^	0.0649	0.0005	120	0.9175	91.75
308	Blank	1.0718	0.0095	2280	–	–
	1 × 10^–5^	0.5982	0.0053	1272	0.4419	44.19
	5 × 10^–5^	0.4689	0.0041	984	0.5625	56.25
	1 × 10^–4^	0.3181	0.0028	672	0.7032	70.32
	5 × 10^–4^	0.2260	0.0020	480	0.7891	78.91
	1 × 10^–3^	0.1659	0.0014	336	0.8452	84.52
318	Blank	1.1868	0.0105	2520	–	–
	1 × 10^–5^	0.6693	0.0059	1416	0.436	43.60
	5 × 10^–5^	0.5435	0.0048	1152	0.542	54.20
	1 × 10^–4^	0.3599	0.0031	744	0.6967	69.67
	5 × 10^–4^	0.2691	0.0023	552	0.7733	77.33
	1 × 10^–3^	0.2119	0.0018	432	0.8215	82.15
328	Blank	1.2423	0.0110	2640	–	–
	1 × 10^–5^	0.711	0.0063	1512	0.4277	42.77
	5 × 10^–5^	0.5839	0.0051	1224	0.5299	53.00
	1 × 10^–4^	0.3921	0.0034	816	0.6844	68.44
	5 × 10^–4^	0.3021	0.0026	624	0.7568	75.68
	1 × 10^–3^	0.2448	0.0021	504	0.8029	80.29

WL measurements were performed at temperatures of 298, 308, 318, and 328 (± 1 K) for X65-steel reaction in HCl (1 M) in the absence and/or presence of various di-imine-SB concentrations (Table 1, C_inh_) to assess the impact of temperature on the IE% of an “inhibitor” candidate and to determine kinetic and thermodynamic parameters. At 1 × 10^–3^ M (C_inh, opt_), the IE% decreased to 80.29% at 328 ± 1 K, the corrosion rate (*CR*) increases at elevated temperatures, owing to the desorption of more di-imine-SB molecules from the surface of X65-steel coupons, lowering the IE%. This behavior is demonstrated experimentally by the evolution of H_2_(g) bubbles on the surface of X65 steel, accompanied by substantial weakening of molecular interactions among di-imine-SB molecules that covers X65-steel surface supporting “physical adsorption”^31,32^.

### Adsorption isotherm

**Figure 2:**
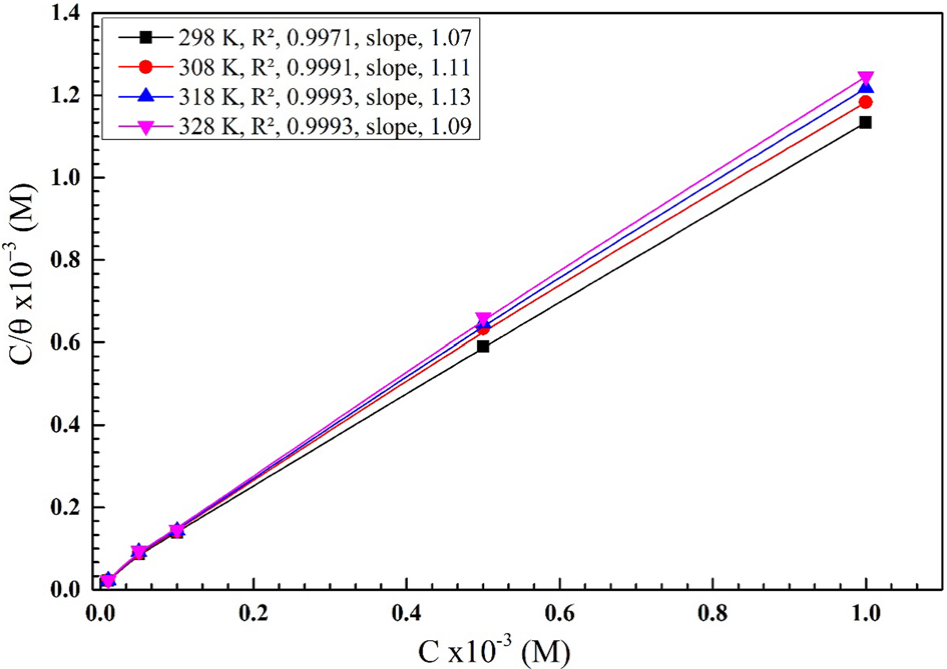
The Langmuir isotherm adsorption plot for X65-steel in 1 M HCl containing different concentrations of the synthesized di-imine-SB compound at different temperatures.

Di-imine-SB's adsorption on X65-steel has a considerable inhibitive effect on HCl offensive action. Therefore, we have considered herein various models of adsorption isotherms “Langmuir, Temkin, Frumkin, Al-Awady, Flory–Huggins and Freundlich”^33^. Nevertheless, the adsorption process depends on metal type, temperature, *θ* “the degree of surface coverage” and the corrosion potential at metal/solution interface^34^. The estimated values of surface coverage were evaluated based on WL data and the correlation coefficient (*R*^2^) at different C_inh_ from 298 to 328 K, in which the adsorption fits Langmuir adsorption isotherm model rather than other models (Table 2). As per the equation^35,36^: $${{\mathrm{C}}_{\mathrm{inh}}}/{\uptheta }=1/{\mathrm{K}}_{\mathrm{ads}}+{\mathrm{C}}_{\mathrm{inh}}$$, where ($${K}_{ads})$$ is the adsorption equilibrium constant, ($${C}_{inh}$$) is the inhibitor molar concentration “di-imine-SB ” and $$\theta$$, attained from the WL as mentioned before^37^. The plot of $${{\mathrm{C}}_{\mathrm{inh}}}/{\uptheta } \,{\text{versus}}\,{\mathrm{C}}_{\mathrm{inh}}$$ from 298–328 ± 1 K produces a straight line and $${K}_{\mathrm{ads}}$$ values were determined from the intercept (Fig. 2, Table 3) in which both *R*^2^ and slope are close to unity which favors Langmuir’s adsorption isotherm. As the temperature exceeded 328 K, the rate of inhibitor desorption was higher than the rate of its adsorption. At 318 K, water adsorption outpaced water desorption, indicating that di-imine-SB molecules were strongly adsorbed to the surface of X65 steel^38^. Moreover, $${\Delta G}_{ads}^{^\circ }=-RT ln(55.5{K}_{ads})$$ ^39^, $${\Delta G}_{ads}^{^\circ }$$ is the standard Gibbs free energy of adsorption, *R* is the universal gas constant (8.314 J mol^−1^ K), T is the absolute temperature (± 1 K), and 55.5 represents the molar concentration of water in solution. The calculated values of $${\Delta \mathrm{G}}_{ads}^{^\circ }$$ for di-imine-SB within 298–328 K are recorded in Table 3, all has negative numerical values which indicates a spontaneous adsorption process^40^. When $${\Delta G}_{ads}^{^\circ }$$ ≤ − 20 kJ mol^−1^, favoring dual electrostatic interaction between inhibitor molecules and metal surface “physical adsorption”, while the values nearby − 40 kJ mol^−1^ or higher suggests N_LP_ donation/interactions of the “inhibitor” to metal surface a “chemisorption adsorption”^41^. Thus, − 40 kJ mol^−1^ > $${\Delta G}_{ads}^{^\circ }$$  > − 20 kJ mol^−1^ indicate physicochemical adsorption but the chemisorption process is dominant^41,42^. Similarly, the temperatures declare the enhanced the numerical values of − $${\Delta G}_{ads}^{^\circ }$$ upon rising it, favoring the adsorption of di-imine-SB inhibitor molecule on X65-steel surface (Table 3).

**Table 2:** Output isotherm parameters for di-imine-SB adsorption on X65-steel/HCl(1 M) surface interface using different models at 298 K.

Isotherm model	Parameters	R^2^	K_ads_ (M^−1^)	ΔG°_ads_ (kJ mol^−1^)
Langmuir	Slope, 1.078	0.999	31.40	− 35.605
Freundlich	1/n, 0.149	0.971	0.379	− 7.548
Temkin	A, 0.228	0.979	0.843	− 9.530
Flowry-Huggins	X, 1.998	0.907	0.695	− 9.049
El-Awady	1/y, 1.867	0.948	4.729	− 12.253
Frumkin	Z, − 1.941	0.866	0.394	− 7.644

**Table 3:** Adsorption thermodynamic parameters of the X56-steel surface in 1 M HCl solution containing different concentrations of the synthesized di-imine-SB at various temperatures in kelvin.

Temp. ± 1 K	Slope	R^2^	K_ads_ (M^−1^) × 10^3^	ΔG°_ads_ (kJ mol^−1^)	ΔH°_ads_ (kJ mol^−1^)	ΔS°_ads_ (kJ mol^−1^ K^−1^)
298	1.07	0.9990	31.40	− 35.605	− 12.6657	119.439
308	1.11	0.9991	38.28	− 37.307		121.128
318	1.13	0.9993	39.19	− 38.581		121.324
328	1.09	0.9993	38.88	− 39.772		121.256

### Thermodynamic parameters

#### Adsorption Thermodynamic parameters

**Figure 3:**
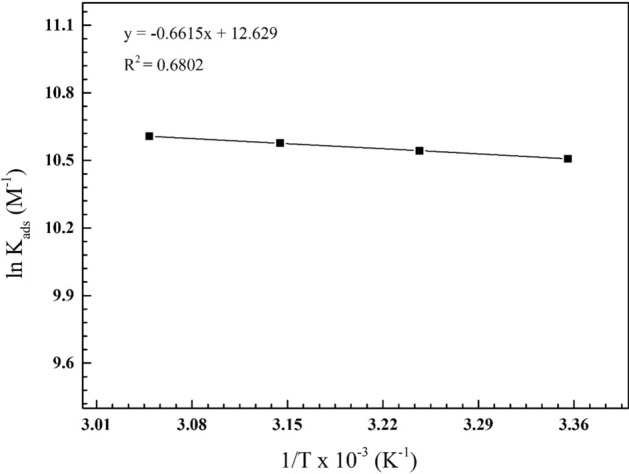
The relation between ln K_ads_ and 1/T for the X65-steel in 1 M HCl medium containing different concentrations of the di-imine-SB inhibitor that applied at different temperature values.

Aided by Van’t Hoff equation^43^: $\mathrm{ln}{\mathrm{ K}}_{\mathrm{ads}}=-{\Delta \mathrm{H}}_{\mathrm{ads}}^{^\circ }/\mathrm{RT}+\mathrm{constant}$*;* we can determine thermodynamic parameters that controls the adsorption of di-imine-SB “inhibitor” on the surface of X65-steel in 1 M HCl solution. Figure 3 illustrates the plot of $\mathrm{ln}{ K}_{\mathrm{ads}}$ (M^−1^) versus 1/T (K^−1^), the heat of adsorption ${(\Delta H}_{\mathrm{ads}}^{^\circ }$) can be obtained from the slope $(-\frac{{\Delta \mathrm{H}}_{\mathrm{ads}}^{^\circ }}{\mathrm{ R}})$. Whereas, the standard adsorption entropy $({\Delta \mathrm{S}}_{ads}^{^\circ })$ can be inferred from: ${\Delta \mathrm{S}}_{ads}^{^\circ }={\Delta \mathrm{H}}_{ads}^{^\circ }-{\Delta \mathrm{G}}_{ads}^{^\circ }/\mathrm{T}$^44^, the calculated ${\Delta \mathrm{H}}_{ads}^{^\circ }$ and ${\Delta \mathrm{S}}_{ads}^{^\circ }$ values are listed together in Table 3. The calculated ${\Delta \mathrm{H}}_{ads}^{^\circ }$< 0, thus either “exothermic adsorption” physisorption or chemisorption process, otherwise both sorption’s materialize with different extent. Also, ${\Delta \mathrm{S}}_{ads}^{^\circ }$ are numerically positive (Table 3) causing disorder increases which are the driving force for effective adsorption of di-imine-SB on X65-steel surface in acidic solution and the replacement of adsorbed water molecules by inhibitor molecules^45^.

#### Activation thermodynamic parameters

**Figure 4:**
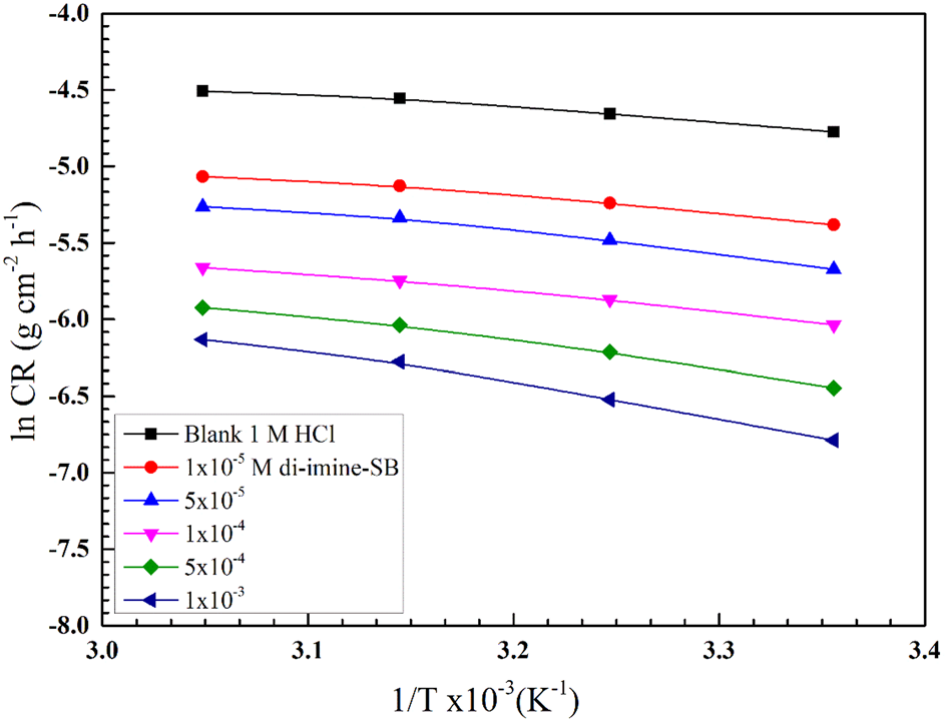
Arrhenius plots, variation of ln CR against 1/T, for X65-steel in 1 M HCl devoid of and containing various concentration of di-imine-SB compound.

**Figure 5:**
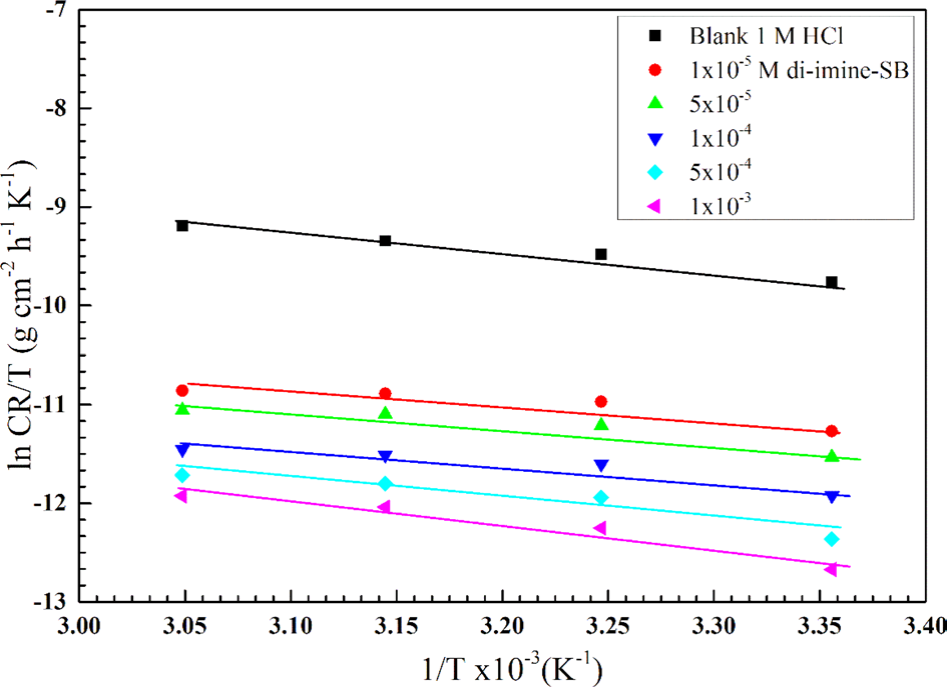
Transition state relation of ln (CR/T) against 1/T, for X65-steel in 1 M HCl devoid of and containing various concentration of di-imine-SB compound.

The mechanism of corrosion inhibition and the properties of the inhibitor under experimental settings must be clarified using the kinetic model. Based on the values of corrosion rate ($CR$) derived from WL measurements (Section "Adsorption isotherm"), the activation energy (${\mathrm{E}}_{a}$), enthalpy- (${\Delta \mathrm{H}}_{a}$), and entropy- of activation ($\Delta {\mathrm{S}}_{a}$) were estimated from Arrhenius and transition state equation^46^: $\mathrm{ln}\,CR=\mathrm{ln}K- ({{E}_{a}}/{RT})$, where $(K)$ is the Arrhenius constant. ${\mathrm{E}}_{a}$ could be obtained from the slope ($-{\mathrm{E}}_{a}$/$\mathrm{R}$) of linear relationship (ln $CR$ vis 1/T) in 1 M HCl without (blank) and with different concentrations “di-imine-SB” inhibitor (Fig. 4). When compared to lower levels of the blank (untreated solution), it is evident that E_a_ is directly proportional to [di-imine-SB], which is attributed to the development/thickening of an electric double layer^47^. Moreover, the metal dissolution reaction requires higher activation energies, hence the entire adsorption process is activation-controlled (Table 4) which is confirmed by a slight decrease in $\mathrm{IE \%}$ upon increasing the temperature, such behavior suggests physical adsorption^48^. From the slope and intercept, (${\Delta \mathrm{H}}_{a}$) and ($\Delta {\mathrm{S}}_{a}$) were calculated, respectively by plotting of ln (*CR*/T) vis 1/T out of the following transition state equation, ${\Delta \mathrm{H}}_{a}$ and $\Delta {\mathrm{S}}_{a}$ were calculated, $\mathrm{ln}\,{\mathrm{CR}}/{\mathrm{T}}={-{\Delta \mathrm{H}}_{\mathrm{a}}}/{\mathrm{RT }}+\mathrm{ln}{\mathrm{R}}/{\mathrm{Nh}}+{\Delta {\mathrm{S}}_{\mathrm{a}}}/{\mathrm{R }},$ where $(N)$ is Avogadro's number and $(h)$ is Planck’s constant, (Fig. 5, Table 4). Endothermic dissolution process of X65-steel is expected when (${\Delta \mathrm{H}}_{a}$) > 0, endorsing difficult dissolution^49^ of X65-steel in presence of di-imine-SB “inhibitor”. The large and imaginary values of activation entropies ($\Delta {\mathrm{S}}_{a}$) in both inhibited/uninhibited aqueous di-imine-SB (Table 4), favor associated- rather than dissociated- activated complex (the rate-determining step), owing to disordering decrease takes place from reactants^50^.

**Table 4:** Activation thermodynamic parameters for corrosion of X65-steel in 1 M HCl in absence and presence of different concentration di-imine-SB at different temperatures.

Conc. (M)	E_a_ (kJ mol^−1^)	ΔH_a_ (kJ mol^−1^)	ΔS_a_ (kJ mol^−1^ K^−1^)
Blank	12.0777	9.4801	− 253.793
1 × 10^–5^	13.3866	10.7890	− 254.432
5 × 10^–5^	15.2480	12.6504	− 250.369
1 × 10^–4^	14.9046	12.3070	− 254.774
5 × 10^–4^	19.7318	17.1342	− 242.104
1 × 10^–3^	34.7488	32.1512	− 197.291

### Potentiodynamic polarization measurements (PDP)

**Figure 6:**
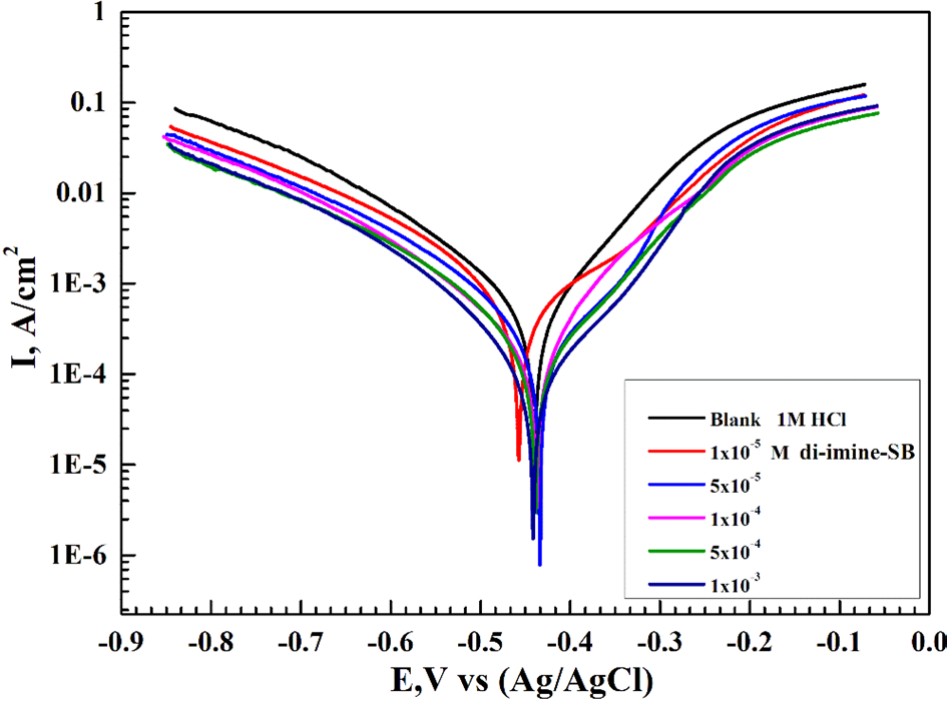
Potentiodynamic polarization curves for X65-steel in 1 M HCl in absence and presence of different concentration of di-imine-SB at 298 K.

In order to determine the cathodic and anodic polarization curves for X65-steel corrosion in 1 M hydrochloric acid with/without di-imine-SB at 298 K, the PDP approach was used after holding the electrodes at E_opc_ for 30 min. Nevertheless, the PDP findings support the WL acquired information. Meanwhile, we can account for corrosion potential (${E}_{\mathrm{corr}}$), specific corrosion current density (${i}_{\mathrm{corr}}$), polarization resistance (${R}_{\mathrm{p}}$), anodic (*β*_a_) and cathodic (*β*_a_) Tafel slopes by extrapolating anodic/cathodic Tafel lines. The $\theta$ and IE% values, were derived from: $\uptheta =(1-{\mathrm{i}}_{\mathrm{corr}}/{\mathrm{i}}_{\mathrm{corr}}^{^\circ } )$ and $\mathrm{IE\%}=(1-{\mathrm{i}}_{\mathrm{corr}}/{\mathrm{i}}_{\mathrm{corr}}^{^\circ } )\times 100$^51,52^, where, ${i}_{\mathrm{corr}}/{i}_{\mathrm{corr}}^{^\circ }$ symbolize the values of specific corrosion current density with/without di-imine-SB “inhibitor”, respectively (Table 5). While ${R}_{\mathrm{p}}$ was estimated from “Stern–Geary” equation: ${\mathrm{R}}_{\mathrm{p}}={\upbeta }_{\mathrm{a}}{\upbeta }_{\mathrm{c}}/2.303 {\mathrm{i}}_{\mathrm{corr}} ({\upbeta }_{\mathrm{a}}+{\upbeta }_{\mathrm{c}} )$^53,54^. The effect of increasing C_inh_ molecules on the anodic/cathodic Tafel lines (E _vs._ I) of X65-steel in 1 M HCl at 298 K is displayed in Fig. 6. Due to the anodic/cathodic polarization curves being shifted to more noble orientations in comparison to the blank, the presence of "di-imine-SB" results in a drop in both i_corr_ and CR (Fig. 6). Note that, the variability in Tafel slopes (*β*a, *β*c) wasn’t tangible, so the addition of di-imine-SB has no effect on the reaction mechanism. Adding di-imine-SB" to the anodic and cathodic areas within ± 85 mV results in no discernible change in E_corr_ compared to those of the blank one, indicating a mixed-type inhibitor^55,56^. The minor shift in ΔE values supports the geometry blocking active sites paradigm^57^ inhibitory effect of di-imine-SB in agreement with potential time graphs (Supplementary Fig. S2) for X65-steel in the absence/presence di-imine-SB inhibitor, in which a steady state potential attained after 30 min of immersion. For polarization resistance, increasing the values of ${R}_{\mathrm{p}}$ were returned to the adsorption of di-imine-SB molecules through the dissociation of X65-steel owing to their blocking active centers effect. At C_inh, opt_ (1 × 10^–3^ M), 90% IE values intended from the i_corr_ (Table 5), thus “di-imine-SB” inhibitor is found effective to reduce the surface corrosion of X65-steel in 1 M HCl by its adsorption via active sites otherwise by covering/coating the electrode surface^57^.These results agree with WL measurements, that dominates "di-imine-SB" over other known inhibitors under the same conditions^58–62^ (Table 6).

**Table 5:** Tafel parameters for X65-steel in 1 M HCl in absence and presence of di-imine-SB employing different concentrations at 298 K.

Conc. (M)	− E_corr_, (V)	i_corr_		β_a_ (mV dec^−1^)	− β_c_ (mV dec^−^)	CR (mpy)	R_P_ (Ω)	θ	IE %
		(µA cm^−2^)	(A m^−2^)						
Di-imine-SB Inhibitor									
Blank	0.439	800.45	8.0045	111.21	186.07	366.39	37.76	–	–
1 × 10^–5^	0.457 0.433	533.20	5.3320	101.50	165.09	244.06	51.19	0.3338	33.38
5 × 10^–5^		271.00	2.7100	85.40	156.30	124.04	88.49	0.6614	66.14
1 × 10^–4^	0.437	248.53	2.4853	109.61	150.70	113.76	110.89	0.6895	68.95
5 × 10^–4^	0.438	156.46	1.5646	89.83	146.23	71.61	154.46	0.8045	80.45
1 × 10^–3^	0.441	79.83	0.7983	90.12	142.40	36.54	300.20	0.9002	90.02

**Table 6:** Comparison between the corrosion inhibition efficiency of the prepared di-imine-SB inhibitor and some other reported inhibitor for Fe-alloys in acidic media (1 M HCl) using PDP.

No	Inhibitor	Optimum concentration	IE%	References
1	Di-imine-SB	1 × 10^−3^ M	90	Present study
2	Benzylidene Schiff bases (2)	1 × 10^–3^ M	87	^58^
3	(DBB)	5 × 10^–4^ M	84.39	^59^
4	4-PCPTC	1 × 10^–3^ M	89	^60^
5	(4-BAB)	1 × 10^–3^ M	85.7	^61^
6	L3	1 × 10^–3^ M	75.5	^62^

### Electrochemical impedance spectroscopic study (EIS)

**Figure 7:**
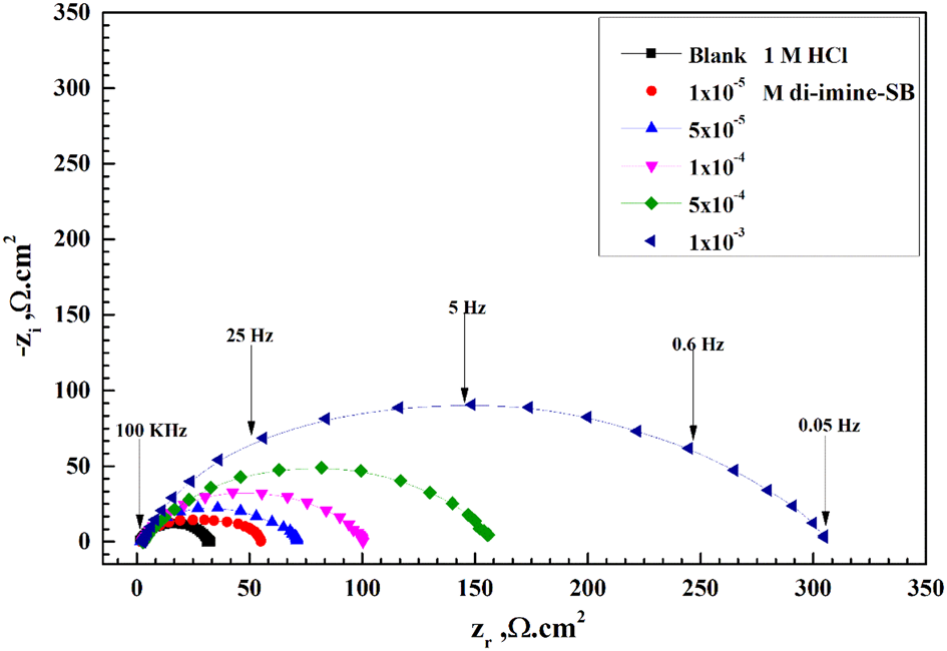
Nyquist diagram for X65-steel in 1 M HCl in absence and presence of different concentration of di-imine-SB at 298 K.

**Figure 8:**
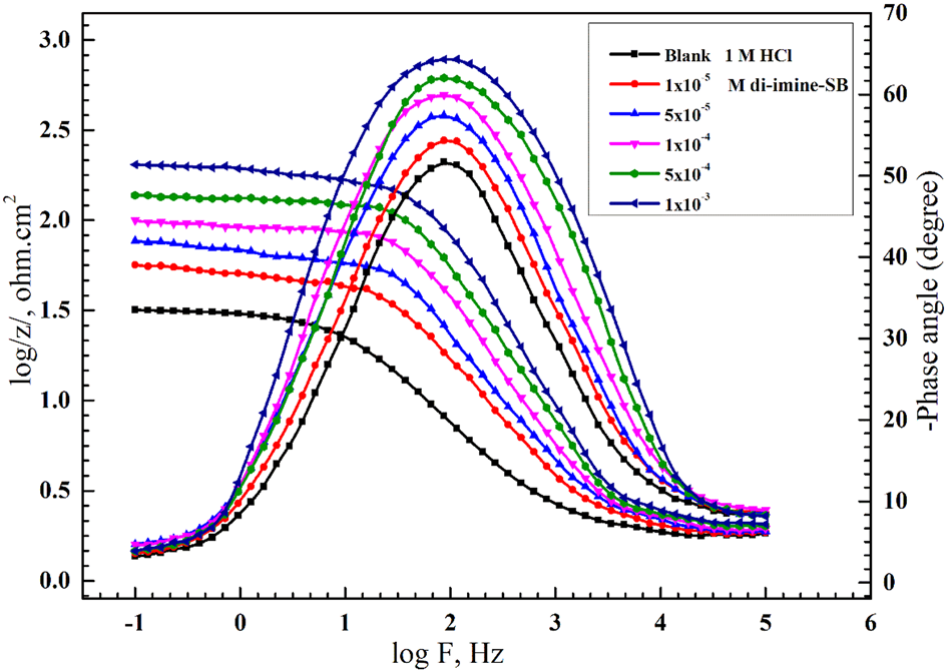
Bode-phase degree diagram for X65-steel in 1 M HCl in absence and presence of different concentration of di-imine-SB at 298 K.

Figures 7 and 8 respectively represent Nyquist and Bode plots for X65-steel corrosion in 1 M HCl solution without/with several C_inh_ “di-imine-SB” after 30 min. immersion at 298 ± 1 K. Inspecting Fig. 7 we can conclude that, the center of the Nyquist impedance curves didn't appear to be a perfect semicircle; instead, it was depressed. This can be endorsed to the frequency dispersion phenomenon owing to the roughness and heterogeneity of the X65-steel surface^63,64^. The Nyquist plots include a single capacitive loop with a single time constant in the Bode-phase plot, hence the reaction of X65-steel in 1 M HCl is controlled by charge transfer process^65^. Additionally, as the number of "di-imine-SB" molecules in the capacitive loop rises (C_inh_), the width of the loop also grows, resulting the CR to drop due to surface coverage of the X65-steel, by the adsorbed di-imine-SB layer^66^. Adding di-imine-SB inhibitor resulting layered adsorption on the X65-steel surface shift the impedance modulus/Z/to higher values and phase angle toward − 90° employing lower and intermediate frequency regions^67^, refer to Bode-phase angle plot (Fig. 8).

**Figure 9:**
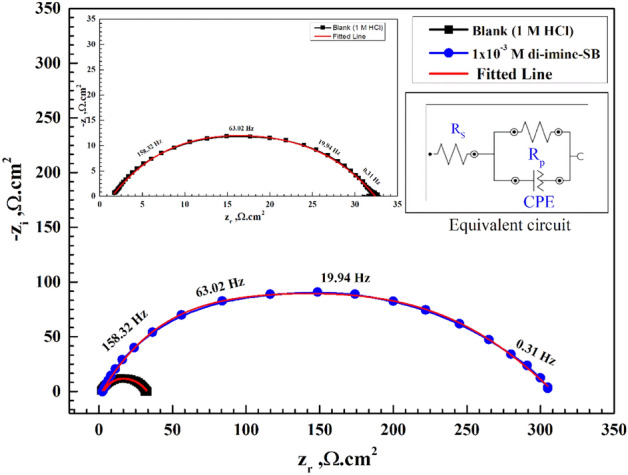
Nyquist plots for X65-steel in 1 M HCl in absence and presence of optimized, 1 × 10^–3^ M, concentration of di-imine-SB (representative one) using the proposed equivalent circuit.

The X65-steel reaction mechanism is unaffected by the presence of di-imine-SB since the shape of EIS spectra (Nyquist and Bode) does not alter. The inset of Fig. 9 suggests an outstanding simulation of "the best equivalent circuit" that fits the EIS data in the absence/presence of "di-imine-SB" at the optimum concentration (C_inh_, opt) of 1 × 10^–3^ M including solution resistance (${R}_{\mathrm{s}})$, constant phase element (CPE) and polarization resistance $\left({R}_{\mathrm{p}}\right)$^68–70^. The ideal CPE can replace the frequency dispersion-prone CPE (*C*_dl_) to produce simulated data that is better suited^71^. With the aid of EIS spectral data fits, the electrochemical parameters and the IE% values were derived from the equation: $\mathrm{IE\%}=(1-{\mathrm{R}}_{\mathrm{p}(\mathrm{blank})}/{\mathrm{R}}_{\mathrm{p}(\mathrm{inh}.)})\times 100$^72^, where,${R}_{\mathrm{p}(\mathrm{blank})}$ and ${R}_{\mathrm{p}(\mathrm{inh}.)}$ are the polarization resistances of X65-steel without/with “di-imine-SB”, respectively. Whereas, the double-layer capacitance $({C}_{\mathrm{dl}})$ is derived from: ${\mathrm{C}}_{\mathrm{dl}}=1/(2\uppi {\mathrm{R}}_{\mathrm{p}}{\mathrm{F}}_{\mathrm{img}\to \mathrm{Max}} )$^73^, where, $F$($\mathrm{img}\to \mathrm{Max}$), is the frequency at maximum imaginary resistance (Table 7). Moreover, the values of (${R}_{\mathrm{p}}$) is directly proportional to the added di-imine-SB compared to the blank, while (${C}_{\mathrm{dl}}$) values show an opposite trend (Table 7). The measured ${R}_{\mathrm{p}}$ value (52.03 cm^2^) at 1 × 10^–5^ M compared to blank (30.31 cm^2^) reaches a maximum at 301 cm^2^ with 1 × 10^–3^ M di-imine-SB inhibitor, representing the greatest increase in surface coverage. The adsorption of “di-imine-SB” molecules on X65-steel surface causes desorption of water molecules, thus the protective layered “di-imine-SB” diminishes the amount of aqueous HCl being in contact with the surface, lowering the steel corrosion rate^74^. The IE% of di-imine-SB verses X65-steel in 1 M HCl exceeds 90% which was supported by WL and PDP measurements. This is connected to the –C=N group, hetero atoms, benzene ring and electron donating groups (CH_3_) in the di-imine-SB structure, in which the methyl moiety increases the ED on the nitrogen’s, ensuing high IE%^75^.

**Table 7:** Electrochemical impedance spectroscopy parameters for X65-steel in 1 M HCl in absence and presence of different concentrations of di-imine-SB at 298 K.

Inhibitor	Conc. (M)	R_s_ (Ω cm^2^)	R_ct_ (Ω cm^2^)	n	C_dl_ (F cm^−2^) × 10^–5^	θ	IE %
Di-imine-SB	Blank	1.549	30.31	0.803	4.177	–	–
	1 × 10^–5^	1.946	52.03	0.772	3.853	0.4176	41.76
	5 × 10^–5^	1.634	69.21	0.803	3.533	0.5622	56.22
	1 × 10^–4^	1.416	98.45	0.783	1.284	0.6922	69.22
	5 × 10^–4^	1.071	152.03	0.764	5.248	0.8006	80.06
	1 × 10^–3^	1.587	301.00	0.791	1.672	0.8993	89.93

### Computational part

**Figure 10:**
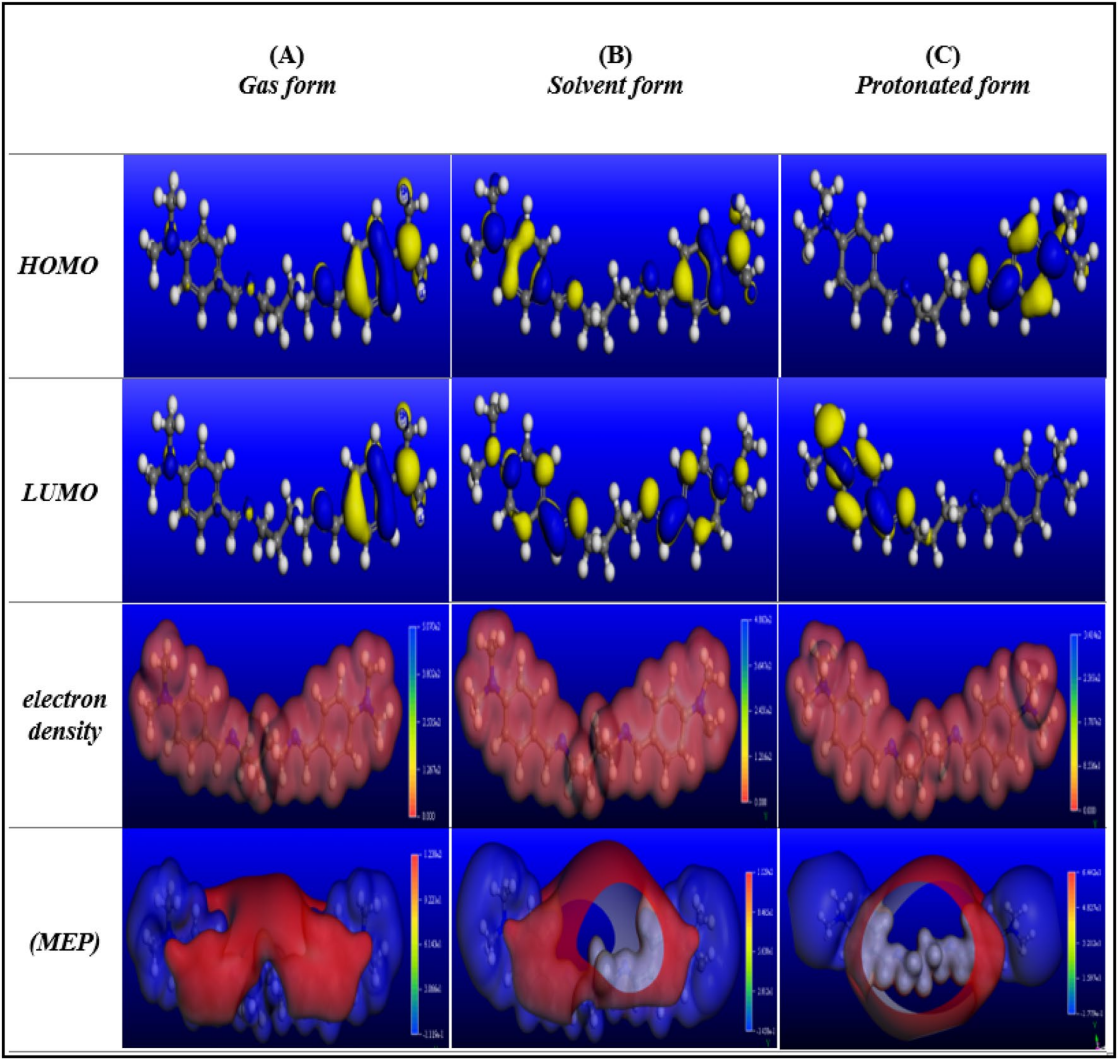
The (HOMO), (LUMO) total occupation, total electron density and molecular electrostatic potential (MEP) mapping distribution for the investigated di-imine-SB inhibitor in (**A**) gas, (**B**) solvent, (**C**) protonated form. Structures were extracted from Dassault Systems Materials Studio Tutorials, BIOVIA, 5005 Wateridge Vista Drive, San Diego, CA 92121, USA, 2017.

DFT is acknowledged to be a powerful approach for predicting the chemical/structural reactivity of an inhibitor candidates^28^. Aided by Koopman’s theorem has been used to target, ${E}_{\mathrm{HOMO}}$, ${E}_{\mathrm{LUMO}}$ and ΔE_gap_ = ${E}_{\mathrm{LUMO}}$ − ${E}_{\mathrm{HOMO}}$ in eV. The frontier orbital energies can be correlated with various quantum parameters, e.g., ionization potential ($I$), electron affinity ($A$), global hardness ($\eta$), and electronegativity ($\chi$) as per the following equations^76,77^: $\mathrm{A}=-{E}_{\mathrm{LUMO}}$; $\upchi =\frac{-({E}_{\mathrm{HOMO}}+{E}_{\mathrm{LUMO}})}{2}$; $\mathrm{I}=-{E}_{\mathrm{HOMO}}$; $\upeta =\frac{\Delta {\mathrm{E}}_{\mathrm{gap}}}{2}$*,* (Table 8). After successful geometry optimization of the examined di-imine-SB, we carried out HOMO, LUMO, and electron density (ED) calculations in addition to molecular electrostatic potential (MEP) mapping distribution for gaseous, solvated H_2_O, and protonated forms (Fig. 10). The HOMO and the LUMO are located above “active centers” of high ED (imine groups, benzylidene rings and the terminal tertiary amines) which enhance the probability of adsorbing “di-imine-SB” molecules on steel surface. Nevertheless, the ED regions can be detected from MEP map (Fig. 10), the extra negative charge “red region” includes N_LP_ and the benzylidene ring's *π*-electrons (i.e., nucleophiles), promotes di-imine-SB's adsorption on metal surfaces in addition to the d-orbitals of Fe^78^ resulting its adsorption, causing corrosion inhibition. Nevertheless, the aggressive chloride anions are driven away from the metal surface by such adsorption, creating a protective layer that reduces metal corrosion and raises the IE%. An additional local reactivity descriptor is the Fukui function/indices that has been widely applied to either account or understand the interaction/reactivity of an inhibitor towards steel corrosion^79^. The Fukui indices are active sites for nucleophilic and electrophilic attack regarding local reactivity are calculated using the finite mathematical difference approximations^80^: ${f}_{k}^{-}=[{\mathrm{q}}_{\mathrm{k}}\left(\mathrm{N}\right)-{\mathrm{q}}_{\mathrm{k}}\left(\mathrm{N}-1\right)]$ and ${f}_{k}^{+}=[{\mathrm{q}}_{\mathrm{k}}\left(\mathrm{N}+1\right)-{\mathrm{q}}_{\mathrm{k}}\left(\mathrm{N}\right)]$, where ${q}_{k}$ is the charge at atomic center k. While atomic charges can be obtained through different approaches, Hirshfeld charges are the most accurate since it is corrected based on the bond order between atoms. Table.S-2 lists the Fukui indices for di-imine-SB in the gaseous and aqueous phases calculated using the Hirshfeld charges obtained from Materials Studio 6.0 (17.1.0.48) software according to DMol3 module. Observing ƒ^+^ and ƒ^−^ Fukui values (Table S2), the highly positive (ƒ^+^) favor nucleophilic attack in that region/atom, therefore the inhibitor candidate accepts electrons from steel surface in easy manner. On the other hand, the high negative (ƒ^−^) suggest an electrophilic attack in which the inhibitor donate electrons to steel surface^81^. The highest ƒ^+^ located on N_1_, N_6_, C_7_, C_9_ and C_17_; so steel back donation can occur at these sites and those estimated for ƒ^−^ are located on N_14_, N_24_, C_18_ and C_20_ centers providing steel with electrons including N_1_ and N_6_ atoms, hereby mutually involved in both donating and accepting electrons. Meanwhile, the chemical reactivity an inhibitor candidate's often increases with smaller (ΔE_gap_) due to the low excitation energy needed for an electron to be promoted (HOMO → LUMO). Such values were in accordance with those reported by Obot et al.^82^, the computed ΔE_gap_ of 3.01, 2.84, and 0.40 eV for gaseous-, aqueous-, and protonated phases, respectively (Table 8). Moreover, the inhibitor outermost electrons are more polarizable towards d**-**orbitals of iron. Therefore, the di-imine-SB molecules can accept electrons (*E*_LUMO_ will be reduced) from iron d-orbitals based on back-donation concept “synergetic effect” reflecting high adsorption efficiency. On the other hand, ΔE_gap_ is directly proportional to the hardness ($\eta$), the harder the molecule the larger is ΔE_gap_. Nevertheless, the lesser ΔE_gap_, the investigated molecule is more likely soft, accordingly soft molecules are frequently more reactive than hard one^83^. The hardness ($\eta$) values were decreasing in the order: protonated < aqueous < gas, respectively and the *E*_HOMO_ of the protonated di-imine-SB is found higher than those estimated for the solvated and neutral molecules (Table 8). Thus, the protonated form has the highest propensity to donate electrons which favors strong adsorption binds on the surface of X65-steel. Moreover, di-imine-SB (H^+^) has the highest chemical reactivity owing to lowest values of ΔE, $\eta$ and $I$ values, therefore it is more feasible to bind effectively on the surface of X65-steel than other forms^82^. The fraction of electron transference (ΔN) displays the reactivity di-imine-SB is derived from ΔN = (φ_Fe_ − $\upchi$_compd._)/[2 (η_Fe_ + η_compd._)], where the work function (φ) of Fe (1 1 0) plan is 4.82 Ev, refer to Table 8^84^. According to Lukovits’s findings, when ΔN is less than 3.6^85^, the adsorption probability features of the adsorbent increases with growing ΔN value. Also, the positive values of ΔN, indicates the ability of electron(s) transfer from HOMO of di-imine-SB to partially filed Fe 3d-orbitals. Whereas the imaginary back donation energy (*E*_back_) represent the required energy to control the back donation process from 3d-orbitals (Fe) to di-imine-SB^86^. Moreover, the higher the inhibition efficiency (IE%) the higher is the dipole moment (*µ*) as a result of strong dipole–dipole interaction, causing corrosion diminishes on X65-steel surface^41^, refer to Table 8.

**Table 8:** Quantum chemical calculated parameters of the investigated di-imine-SB inhibitor.

Phase	E_HOMO_ (eV)	E_LUMO_ (eV)	Δ E (eV)	A (eV)	I (eV)	X (eV)		Ƞ (eV)		ΔN	µ (Debye)	E_back_
Gas	− 4.077	− 1.066	3.011	1.066	4.077		2.571		1.505	0.746	0.805	− 0.376
Water	− 4.312	− 1.471	2.840	1.471	4.312		2.892		1.420	0.678	0.426	− 0.355
Protonated	− 1.167	− 0.760	0.407	0.760	1.167		0.963		0.203	9.464	5.402	− 0.051

### Monte Carlo simulation (MCs)

**Figure 11:**
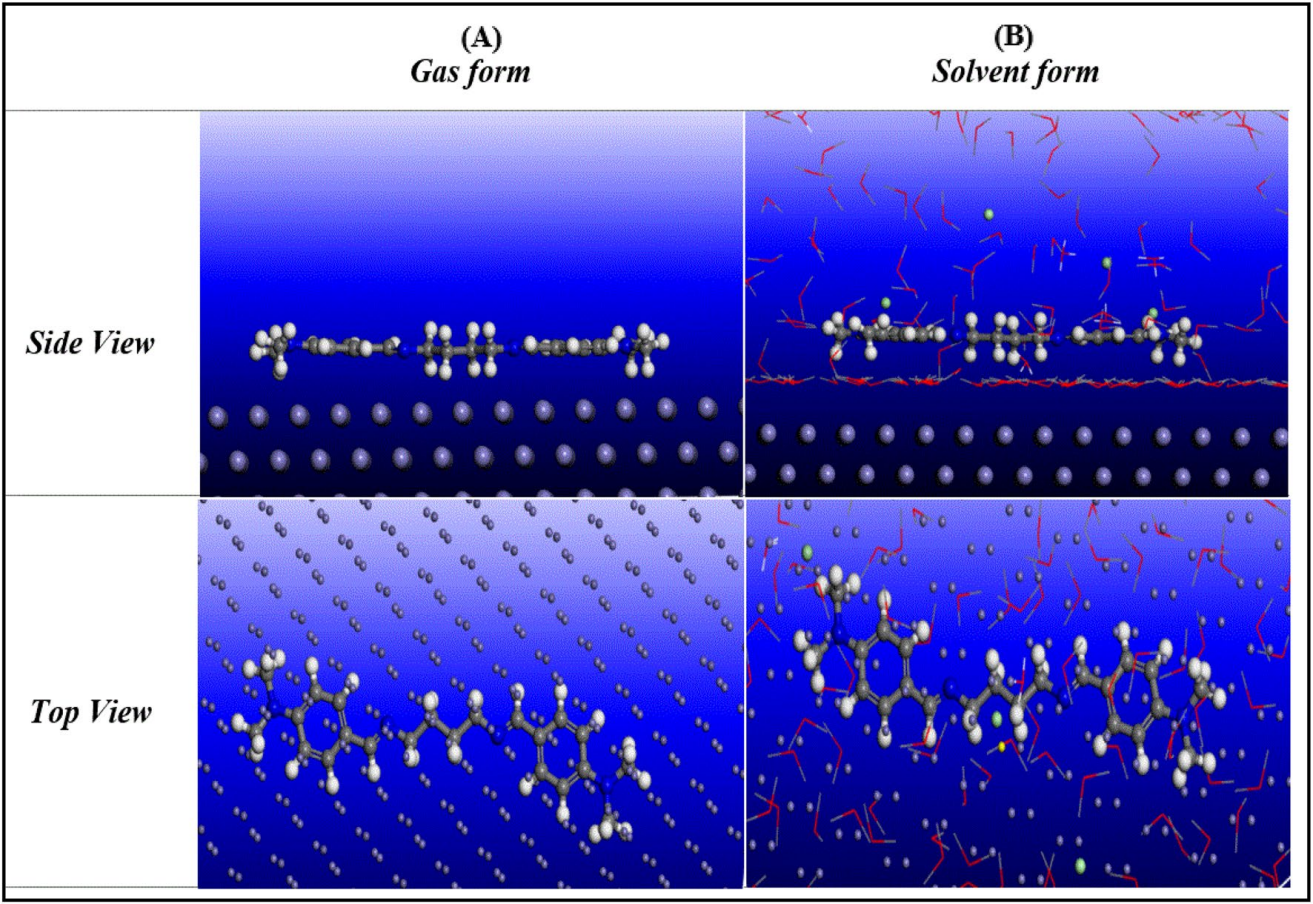
Side and top views of the adsorption mode of di-imine-SB inhibitor molecule in (**A**) gas and -(**B**) simulated acid solution on Fe (1 1 0) substrate, extracted from Dassault Systems Materials Studio Tutorials, BIOVIA, 5005 Wateridge Vista Drive, San Diego, CA 92121, USA, 2017.

Monte Carlo simulation (MCs) is a powerful theoretical tool that can mimic inhibitors molecular interactions with iron metal surfaces, the current computations is similar to that suggested by Singh et al.^87^. Snapshots for the optimized di-imine-SB in gas phase on Fe (1 1 0) cleaved surface are presented in Fig. 11A in addition to that simulated in acidic solution (200 H_2_O molecules, 5 H_3_O^+^ and 5 Cl^−^ ions) to mimic the corrosion media (Fig. 11B) until reaching an equilibrium. The optimized structure side view (snapshots) reveals a strong interactions between flat parallel orientation of the di-imine-SB and the Fe (1 1 0) surface which lead to iron steel surface protection^73^. On the other hand, top views show di-imine-SB molecules covering large surface area of X65-steel surface. Energy of adsorption (E_ads_) is the energy released upon inhibitor binding “relaxed” on metal surface while the rigid adsorption energy (E_rigid_), is the released/required energy for the unrelaxed adsorbate “an inhibitor” to be adsorbed on the adsorbent “Metal surface”, whereas the deformation energy (E_def_) is the released energy from the relaxation of the adsorbed molecules on steel surface (E_ads_ = E_rigid_ + E_def_)^88^. The total Energy (E_tot_) of an inhibitor, is the sum of internal energy and adsorption energy (E_ads_). In fact, the least values of E_ads_ (Table 9) reveal a spontaneous physical adsorption of gaseous- and solvated- di-imine-SB on the X65-steel surface, i.e., more adsorbent molecules are located on the surface in agreement with earlier interpretations^89^. Consistently, E_ads_ of di-imine-SB “inhibitor” is found too low compared to that of H_2_O, H_3_O^+^ and Cl^−^ ions (Table 9). Therefore, it has propensity to progressively replace the adsorbed water molecules to form a thermodynamically stable adsorption layered film on Fe (1 1 0) surface. While E_ads_ show further decrease in presence of water molecules which is associated with intermolecular HB interactions between di-imine-SB and water molecules that enhance its adsorption on the steel surface^90^. Both DFT and MCs calculations favors the feasible application of di-imine-SB as a potential corrosion inhibitor.

**Table 9:** The outputs energies calculated by Monte Carlo simulation for di-imine-SB inhibitor in gas and simulated acid solution phases on Fe (1 1 0).

Compound	E_T_ (KJ mol^−1^)	E_ads_ (KJ mol^−1^)	E_rig.ads_ (KJ mol^−1^)	E_def._ (KJ mol^−1^)	3D	3D H_2_O	3D H_3_O^+^	3D Cl^−^
Di-imine-SB	− 276.72	− 226.52	− 211.42	− 15.11	− 226.53	–	–	–
Di-imine-SB + H_2_O + 5HCl	− 3462.49	− 4621.12	− 3410.05	− 1211.07	− 132.53	− 6.64	− 147.05	− 99.34

### SEM and EDX

**Figure 12:**
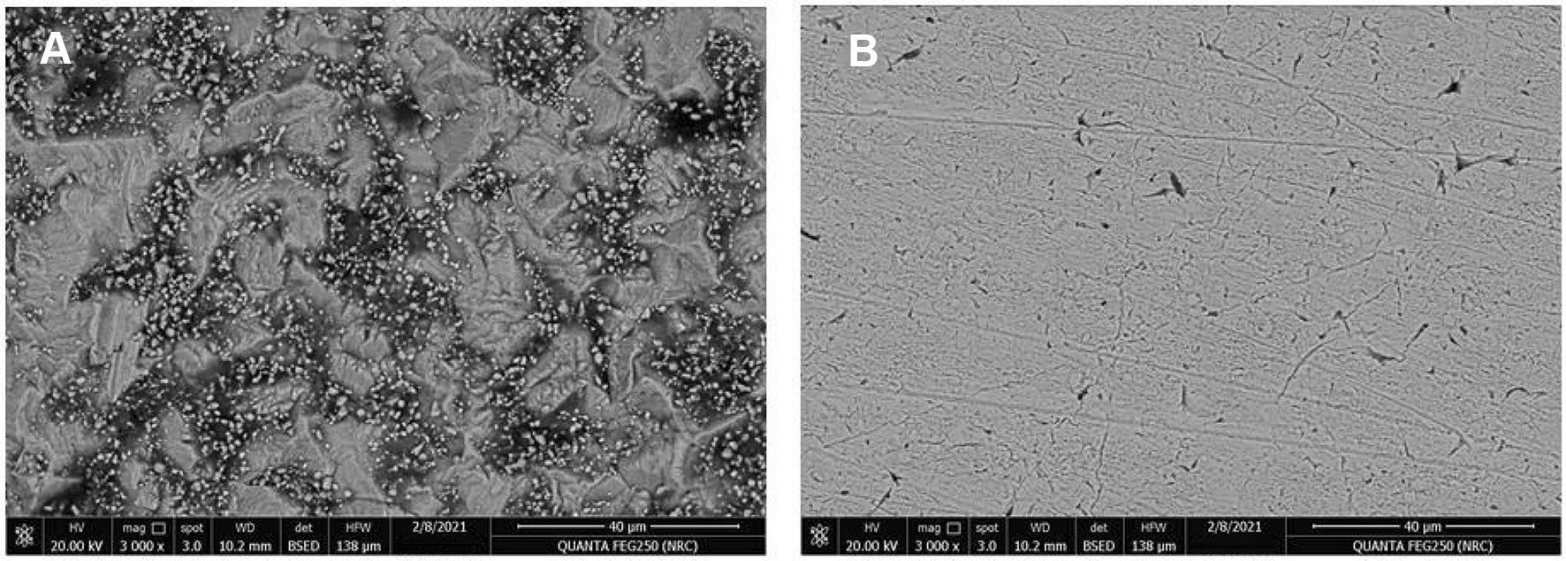
SEM for the X65-steel surface: (**A**) sample immersed in 1 M HCl without di-imine-SB inhibitor, (**B**) sample immersed in (1) M HCl with (1 × 10^–3^ M) of di-imine-SB inhibitor.

**Figure 13:**
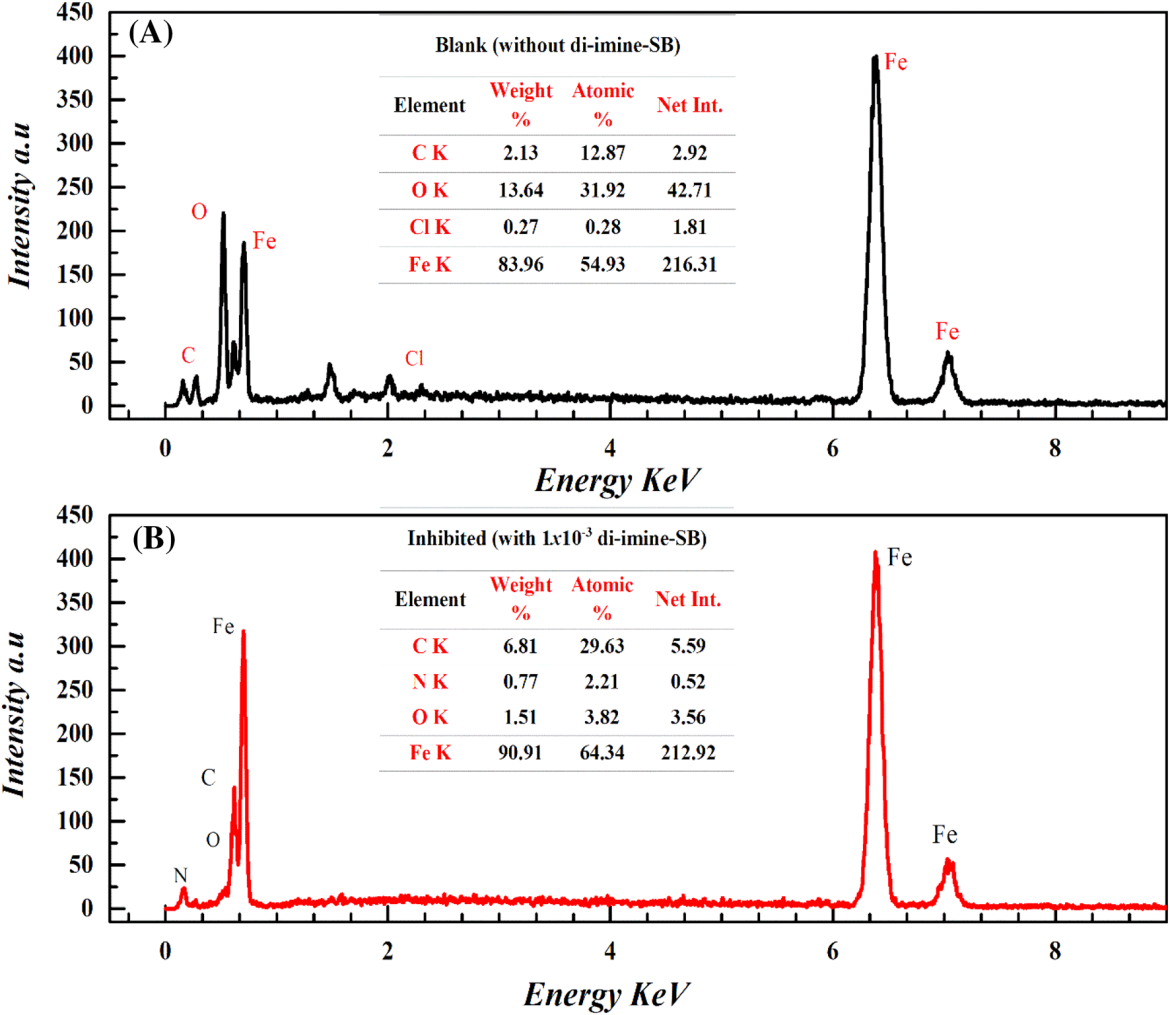
EDX for the X65-steel surface: (**A**) sample immersed in 1 M HCl acid solution without di-imine-SB inhibitor, (**B**) sample immersed in 1 M HCl acid solution with (1 × 10^–3^ M) of di-imine-SB inhibitor.

The displayed SEM (Fig. 12) and EDX (Fig. 13) measurements for X65-steel sheets (1 × 1 × 0.1) cm^3^ immersed for 6* h* in 1 M HCl (blank) and those treated with 1 × 10^–3^ M di-imine-SB “inhibitor” at the same conditions. Analyzing the SEM images in the absence of di-imine-SB “inhibitor”, an irregular rough damaged X65-steel surface is observed owing to steel dissolution in 1 M HCl corrosive media (Fig. 12a). On the other hand, the lumpiness was absent, and a smooth X65-steel surface was evidenced after being treated with 1 mM of di-imine-SB which provides good protection against corrosion and lowering its rate (Fig. 12b). The EDX spectra Fig. 13a,b configure the elemental and aqueous components on the outer layer surface of X65-steel before and after being in contact with di-imine-SB (1 mM). The appearance of the nitrogen atom (N) peak and increasing the C-content ensures the adsorption of di-imine-SB on the steel surface forming a protected area of the adsorbed inhibitor, which reduces the corrosion. These outcomes are also supported by the decrease of chlorine and oxygen peak intensities. increasing the intensity of Fe-peak confirms the reduction of metal oxide formation due to the HCl severity as well^91^. EDX study showed in Fig. 13a,b confirms the SEM observations and found consistent with WL, PDP and EIS results.

### The proposed di-imine-SB inhibition mechanism

**Figure 14:**
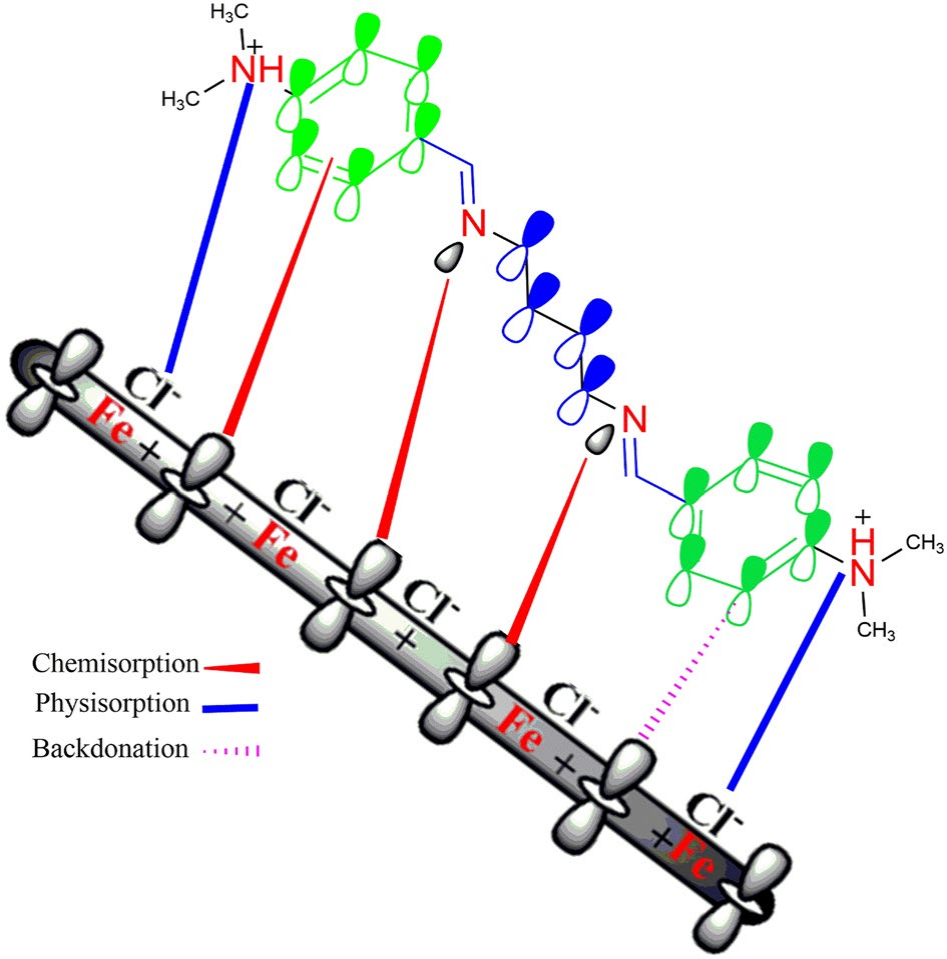
Suggested adsorption model of the prepared di-imine-SB inhibitor over the X-65 steel.

The di-imine-SB "Schiff base" investigated herein is structurally featured with imine (–CH = N–) functionals, delocalized aromatic π electrons in addition to –N(Me)_2_ moieties that may interact with X65-steel surface. Firstly, few nucleophiles active centers (N^+^) get protonated into the acidic medium (1 M HCl) and electrostatically interact with the accumulated chloride (Cl^−^) ions nearby the metal-solution interface, therefore represent physical adsorption. After partial metal dissolution, the unoccupied d-orbitals that accept(s) *lone-pair* electrons; thus, gets coordinated with nitrogen through imine functional group(s), suggests chemical adsorption. Nevertheless, the expected back donation results from π electrons delocalization’s/interaction’s with X65-steel. The current results support mixed physical/chemical adsorption (Fig. 14) which found consistent with other molecules containing benzylidene and imine groups in addition to hetero-atoms^92–94^.

## Conclusion

The di-imine-SB compound has enriched with electron donating groups that support its role as X65-steel corrosion inhibitor. It decreases the weight loss of X65-steel to 0.0649 g with inhibition efficiency 91.75% at 298 K. Adsorption behavior of di-imine-SB followed Langmuir adsorption model. ${\Delta G}_{ads}^{^\circ }$ value (− 35.605 kJ mol^−1^) indicated the physicochemical adsorption of di-imine-SB on the X65-steel surface; however, chemisorption process is a bit dominant. di-imine-SB dropped the I_corr_ of X65-steel from 800.45 µA cm^−2^ to 79.83 µA cm^−2^ as it shielded the active centers of X65-steel away from HCl offensive action. Also, di-imine-SB compound decreased the capacitance of the double layer at the HCl/X65-steel interface to 0.1672 µF cm^−2^ due to the higher resistance of protective layer formed at HCl/X65-steel interface. Thermodynamic parameters of X65-steel reaction confirmed the protective effect of di-imine-SB compound. SEM analysis indicated the smoothness appearance of X65-steel surface after adding 1 mM of di-imine-SB, and this in agreement with the experimental data. The experimental results were supported by DFT and MC simulation calculations, which also provided deep insight into the corrosion inhibition process.

## Supplementary Information

Supplementary Information.

## Data Availability

All data generated or analyzed during this study are included in this manuscript.
