# Supplementary material for: Electrochemical, surface analysis, computational and anticorrosive studies of novel di-imine Schiff base on X65 steel surface
Source: Sci Rep. 2023 Jun 28;13:10457. doi: 10.1038/s41598-023-37321-8 (PMC10307884; doi:10.1038/s41598-023-37321-8)
Supplement: Supplementary file 1 — Supplementary Information. [file 41598_2023_37321_MOESM1_ESM.docx]

|  |  | Weight Loss | | PDP | | EIS | |  |
| --- | --- | --- | --- | --- | --- | --- | --- | --- |
|  | ***Conc.***  ***(M)*** | CR  (g/cm^2^.h) x10^4^ | **±** SD.DEV  x10^-4^ | i_corr_ (µA/cm²) | **±** SD.DEV | R_ct_ (Ω.cm^2^) | **±** SD.DEV |  |
| *Blank* | *0.00* | 69 | 4.063 | 800.45 | 3.904 | 30.31 | 2.823 | |
| *di-imine-SB Inhibitor* | *1x10-5* | 38 | 4.036 | 533.20 | 2.260 | 52.03 | 3.148 | |
|  | *5x10-5* | 29 | 4.359 | 271.00 | 2.594 | 69.21 | 2.679 | |
|  | *1x10-4* | 19 | 4.100 | 248.53 | 2.551 | 98.45 | 2.423 | |
|  | *5x10-4* | 12 | 4.106 | 156.46 | 2.260 | 152.03 | 3.211 | |
|  | *1x10-3* | 5 | 3.612 | 79.83 | 2.562 | 301.00 | 3.751 | |

**Table.S-1:** Standard deviation of output Weight loss, PDP and EIS data for X65-steel electrode immersed in 1 M HCl in absence and presence of different concentrations of the synthesized di-imine-SB inhibitor at room temperature.

**Table.S-2:**  Calculated Fukui functions for the studied di-imine-SB inhibitor.

| **Atoms** | **Gas phase** | | **Aqueous phase** | |
| --- | --- | --- | --- | --- |
|  | **$f^{+}$** | **$f^{-}$** | **$f^{+}$** | **$f^{-}$** |
| N_1_ | 0.045 | 0.036 | 0.061 | 0.046 |
| C_2_ | 0.008 | 08 0.0 | 0.011 | 0.008 |
| C_3_ | 0.003 | 0.005 | 0.006 | 0.004 |
| C_4_ | 0.005 | 0.006 | 0.007 | 0.004 |
| C_5_ | 0.011 | 0.007 | 0.011 | 0.005 |
| N_6_ | 0.059 | 0.026 | 0.06 | 0.026 |
| C_7_ | 0.06 | 0.013 | 0.062 | 0.013 |
| C_8_ | 0.02 | 0.032 | 0.024 | 0.03 |
| C_9_ | 0.034 | 0.016 | 0.036 | 0.016 |
| C_10_ | 0.022 | 0.025 | 0.02 | 0.024 |
| C_11_ | 0.04 | 0.018 | 0.032 | 0.017 |
| C_12_ | 0.025 | 0.029 | 0.021 | 0.027 |
| C_13_ | 0.027 | 0.019 | 0.027 | 0.017 |
| N_14_ | 0.022 | 0.052 | 0.015 | 0.051 |
| C_15_ | 0.008 | 0.013 | 0.005 | 0.012 |
| C_16_ | 0.009 | 0.013 | 0.005 | 0.012 |
| C_17_ | 0.048 | 0.014 | 0.066 | 0.021 |
| C_18_ | 0.014 | 0.039 | 0.025 | 0.05 |
| C_19_ | .0260 | 0.021 | 0.04 | 0.027 |
| C_20_ | 0.018 | 0.029 | 0.02 | 0.041 |
| C_21_ | 0.033 | 0.021 | 0.037 | 0.026 |
| C_22_ | 0.02 | 0.034 | 0.022 | 0.043 |
| C_23_ | 0.022 | 0.023 | 0.026 | 0.028 |
| N_24_ | 0.019 | 0.059 | 0.022 | 0.072 |
| C_25_ | 0.007 | 0.014 | 0.005 | 0.018 |
| C_26_ | 0.007 | 0.014 | 0.009 | 0.015 |
|  |  |  |  |  |





**Fig. S-1:** (*N^1^Z,N^4^Z)-N^1^,N^4^*-bis(4-(dimethylamino)benzylidene)butane-1,4-diamine (di-imine-SB).





**Fig. S-2**: Potential time graphs for X65-steel in the absence and presence of various concentrations of di-imine-SB inhibitor at room temperature.
